# The mindful resiliency in recovery model: empowering the transcendence of stigma

**DOI:** 10.3389/fpsyg.2024.1460329

**Published:** 2024-10-25

**Authors:** David I. K. Moniz-Lewis

**Affiliations:** Addictive Behaviors and Quantitative Research Lab, Department of Psychology, Center for Alcohol Substance Use and Addiction, University of New Mexico, Albuquerque, NM, United States

**Keywords:** mindfulness, substance use disorder, stigma, addiction, marginalized populations, resilience, mechanisms of change, context

## Abstract

**Introduction:**

Mindfulness-based interventions show unique promise in treating substance use disorders among marginalized populations who face heightened stigma. The Mindful Resiliency in Recovery Model is introduced as a novel theoretical framework articulating how mindfulness training can mitigate the adverse effects of stigma, enhance psychological resilience, and facilitate sustained recovery from addiction.

**Methods:**

The current manuscript synthesizes various models of mindfulness processes, stigma, and substance use disorder recovery to propose an integrated theoretical framework on the promise of mindfulness-based interventions in supporting recovery. Further, the current manuscript draws upon empirical literature to establish preliminary support for the premises and hypotheses of the Mindful Resiliency in Recovery Model concerning the mechanisms influencing the efficacy of mindfulness-based interventions among marginalized individuals.

**Results:**

Preliminary evidence supports the premises of the proposed model. There is evidence to suggest that specific processes like increased present-moment awareness, acceptance, decentering, reappraisal, and savoring may be especially salient in mitigating internalized stigma and fostering resiliency in recovery. There is a need for additional research on these processes, and contextual factors that may moderate their efficacy.

**Discussion:**

The Mindful Resiliency in Recovery Model has significant implications for optimizing mindfulness-based interventions to empower marginalized individuals to transcend stigma and actualize their capacity for wellbeing in substance use disorder recovery. It provides a roadmap for future research on the mechanisms and contextual factors affecting the efficacy of mindfulness-based interventions for marginalized and stigmatized communities. It further offers guidance to clinicians utilizing mindfulness-based interventions to support individuals experiencing stigma.

## Introduction

Substance use disorder (SUD) poses a significant public health challenge, resulting in significant suffering for individuals, families, and communities (Delphin-Rittmon, 2022; Substance Abuse and Mental Health Services Administration, 2019; Lozano et al., 2012). This burden disproportionately affects individuals from marginalized backgrounds, including racial/ethnic minorities and those from lower socioeconomic status backgrounds, who often face additional barriers to treatment and recovery due to systemic inequities and discrimination (Chartier, 2010; Swan et al., 2021; Vaeth et al., 2017). Over the years, numerous empirically validated pharmacological, psychosocial, and behavioral treatments exist for SUD (Delphin-Rittmon, 2022; Marsch and Dallery, 2012). One such class of intervention approaches, mindfulness-based interventions (MBIs), have emerged as one of the most utilized third-wave behavioral treatments for SUD and other co-occurring pathologies (Korecki et al., 2020; Schwebel et al., 2020). Rooted in contemplative practices found within Eastern wisdom traditions like Buddhism, MBIs leverage non-judgmental present-moment awareness to support individuals with SUD in achieving wellbeing and recovery (Bowen et al., 2021; Kabat-Zinn, 2003). Over the last two decades, a plethora of MBIs, such as mindfulness-based relapse prevention, mindfulness-oriented recovery enhancement, and mindfulness-based cognitive therapy, have been developed and disseminated into clinical settings to support recovery from SUD (Korecki et al., 2020; Bowen et al., 2021; Garland, 2013). Importantly, despite variations in treatment design, each MBI is relatively equivalent in its efficacy (Korecki et al., 2020; Schwebel et al., 2020; Chiesa and Serretti, 2014; Li et al., 2017)—a phenomenon not unique to MBIs but found across varied psychological and behavioral treatments for SUD and other psychopathologies (Budd and Hughes, 2009; Magill et al., 2015).

Given the emergence of different MBI treatment packages targeting SUD, and their relative equivalency, there has been a growing movement to shift the focus of MBI research from merely assessing the efficacy of any given MBI to assessing the processes that make MBIs effective (Hayes et al., 2024; Lindsay and Creswell, 2019). Many theoretical frameworks have been proposed that attempt to articulate the most integral processes underlying the efficacy of MBIs—such as the Monitor and Acceptance Theory (Lindsay and Creswell, 2017) and Mindfulness-to-Meaning Theory (Garland et al., 2015). However, none of these theories have been extended to identify *for whom and in what contexts* specific mindfulness processes are most salient.

### Mindfulness-based interventions among diverse, marginalized, and underserved groups

In this light, recent findings suggest that there may be unique benefits of MBIs for SUD among marginalized individuals specifically, particularly when compared to other empirically-supported SUD treatments like cognitive-behavioral therapy (Dela Cruz et al., 2022; Garland et al., 2016; Greenfield et al., 2018; Spears, 2019; Spears et al., 2017; Sun et al., 2022; Witkiewitz et al., 2013). A study by Witkiewitz et al. (2013) found that mindfulness-based relapse prevention was more efficacious than cognitive-behavioral relapse prevention for women with racial and ethnic minority identities involved in the criminal legal system, with those in mindfulness-based relapse prevention reporting significantly fewer drug use days and lower addiction severity at follow-up. In another study that examined individual race/ethnicity and racial group composition as moderators of mindfulness-based relapse prevention outcomes, it was demonstrated that mindfulness-based relapse prevention resulted in fewer drug use days for racial/ethnic minorities compared to non-Hispanic white individuals (Greenfield et al., 2018). Additionally, participants in groups comprising more than half racial/ethnic minority individuals had better substance use outcomes when in mindfulness-based relapse prevention versus cognitive-behavioral relapse prevention (Greenfield et al., 2018). These findings were substantiated by a recent systematic review of third-wave interventions (which include MBIs) for SUD among people of color and collectivist cultures. This review found that in the vast majority of studies, those who received a third-wave treatment showed greater improvements in at least one substance use outcome relative to controls (Dela Cruz et al., 2022). Importantly, however, few of these studies controlled for extenuating demographic factors such as socioeconomic status and other potential confounding variables often associated with racial/ethnic minority status (e.g., access to healthcare). This suggests that the observed benefits may be influenced by these unaccounted-for factors but nonetheless suggest highlight the potential effectiveness of MBIs among marginalized groups.

The unique promise of MBIs in treating SUD among stigmatized, diverse, and underserved populations is particularly compelling given the disproportionate burden of SUD on marginalized populations (Vaeth et al., 2017; Kilian et al., 2023; Lê Cook and Alegría, 2011; Zemore et al., 2018). Individuals from low-socioeconomic and racial/ethnic minority groups experience greater harm at equivalent levels of substance use (Chartier, 2010; Swan et al., 2021; Roche et al., 2015), and are also less likely to have access to, complete, and benefit from high-quality SUD treatment (Alegria et al., 2011; Lee et al., 2024; Rosales et al., 2022). Furthermore, there is a significant lack of representation in studies seeking to validate empirically-supported interventions for SUD more broadly (Chartier, 2010; Boness et al., 2023; Burlew et al., 2021), with this being especially true of trials evaluating the efficacy of MBIs (Korecki et al., 2020; Spears, 2019).

Together, the disproportionate harms of substance use among marginalized groups, the potential heightened efficacy of MBIs among marginalized populations, and the lack of representation in trials evaluating MBIs underscore a necessity for future research that not only includes more diverse samples but also delineates the factors that may explain their potentially unique efficacy for these populations. Failing to account for demographic factors like race, ethnicity, and socioeconomic in studies evaluating the efficacy of MBIs can confound research findings generally and thus effect the clinical implementation and effectiveness of MBIs. Given that marginalized individuals may experience unique stressors, such as discrimination and economic hardship, which can contribute to the development and maintenance of symptoms of SUD, it is essential to empirically examine in research settings how MBIs specifically may foster innate resilience among marginalized individuals. Thus, as the field shifts its emphasis away from focusing primarily on the outcomes of MBIs, to the processes underlying them, it will be crucial to account for whom any given empirically validated process is most salient (Boness and Witkiewitz, 2023). One potential transdiagnostic process tapped by MBIs that may be particularly relevant for underserved groups is that of stigma and its associated emotional hindrances, such as shame (Greene, 2023; Gull et al., 2023; Luoma et al., 2019).

### Stigma, substance use disorder, and mindfulness-based interventions

Stigma is a complex social phenomenon characterized by stereotypes, prejudice, and discrimination toward individuals and groups based on perceived undesirable attributes and accompanying status in social hierarchies (Corrigan and Watson, 2002; Link and Phelan, 2001). Numerous models propose discrete forms of stigma across levels of context (Stangl et al., 2019; Bos et al., 2013). Public stigma refers to the negative beliefs, assumptions, and/or attitudes held by a society or cultural group, whereas self-stigma (also referred to as internalized stigma) refers to the process by which individuals become aware of, agree with, and apply these negative beliefs, assumptions, and attitudes to oneself (Stangl et al., 2019; Corrigan et al., 2011; Crocker and Major, 1989). When internalized, stigma can lead to numerous harms, such as reduced self-esteem, self-efficacy, quality of life, and interpersonal relationships, as well as increased psychological distress and avoidance of help-seeking behavior (Corrigan et al., 2011; Livingston and Boyd, 2010; Luoma et al., 2007; Schomerus et al., 2011). Shame, the central emotional experience associated with internalized stigma, can lead to social withdrawal, pathological rumination, and exacerbation of existing psychological difficulties (Luoma et al., 2019; Corrigan and Watson, 2002; Hatzenbuehler et al., 2013).

While early models of stigma tended to define and differentiate its impacts across two primary levels—public stigma vs. self-stigma (Link and Phelan, 2001; Link et al., 1989)—more recent frameworks offer nuanced depictions of how stigma manifests at varied levels of context, specifically at the levels of the individual, interpersonal, organizational, community, and public policy (Stangl et al., 2019; Bos et al., 2013; Burke et al., 2009). Further, advances in this literature have led to the identification of varied types of stigma existing at each level of context (Bos et al., 2013). For example, internalized and anticipated stigma represents stigma experiences manifesting at the level of the individual; secondary and enacted stigma represents expressions of stigma at interpersonal levels; structural stigma represents systematic biases and discriminatory practices at the level of organizations, communities, and public policies (Stangl et al., 2019; Bos et al., 2013; Sheehan et al., 2017). Additionally, while it was once assumed that individuals with stigmatized identities would inevitably experience internalized stigma (Link et al., 1989; Goffman, 2009), research has demonstrated this is not always so—with many individuals from stigmatized groups exhibiting resiliency despite existing in social systems that perpetuate stigmatizing narratives (Corrigan et al., 2011; Crocker and Major, 1989; Livingston and Boyd, 2010; Gu et al., 2023). The nuance provided by these updated frameworks allows for an improved capacity to understand not only how stigma manifests across each level, but also how these levels and types of stigma interact to compound harms (Stangl et al., 2019). These updated frameworks further highlight where opportunities for intervention and prevention may exist (Stangl et al., 2019; Gronholm et al., 2017).

These nuanced models are especially important for understanding the multi-level effects of stigma on SUD (Witkiewitz and Tucker, 2024). Globally, SUD is one of the most stigmatized psychological diagnoses (Schomerus et al., 2011; Barry et al., 2014) often falsely perpetuating notions of individuals with SUD as being unpredictable, lesser than, and personally at fault for their condition (Corrigan and Shapiro, 2010; Crisp et al., 2000). As a result, many individuals experiencing substance-related concerns face harms specific to SUD stigma, such as social rejection and discrimination in key life domains like housing, employment, and healthcare (Hatzenbuehler et al., 2013; Room, 2005; Van Boekel et al., 2013; Van Boekel et al., 2015). Importantly, individuals with SUD from marginalized communities, such as racial/ethnic minorities and individuals from low socioeconomic backgrounds, may experience even greater harm as a result of holding multiple stigmatized identities (Greene, 2023; Douglass et al., 2023). Structural inequities and social determinants of health can further exacerbate the harms of stigma, leading to greater barriers to accessing and benefiting from SUD treatment (Lê Cook and Alegría, 2011; Lee et al., 2024; Barry et al., 2014; Room, 2005). The so-called “war on drugs,” arguably better referred to as the “war on *people who use* drugs,” has only intensified these issues in nations like the United States by criminalizing substance use and disproportionately targeting marginalized populations, thereby perpetuating stigmatizing narratives and further increasing barriers to treatment and recovery (Earp et al., 2020; Mauer and King, 2007).

Considering the significant role of stigma experiences in exacerbating SUD symptoms and hindering recovery, particularly among marginalized populations, it is essential to identify interventions that can effectively target these processes specifically. Notably, MBIs and related third-wave interventions have shown promise in reducing self-stigma and shame across psychological disorders, including among individuals with SUD (Luoma et al., 2019; Livingston et al., 2012; Luoma et al., 2008; Mehel Tutuk and Budak, 2023; Moore et al., 2022; Stynes et al., 2022). Theory suggests that core processes such as the cultivation of non-judgmental awareness, self-compassion, and psychological flexibility in MBIs may help individuals develop a more balanced and accepting relationship with their experiences (Lindsay and Creswell, 2017; Garland et al., 2015). This, in turn, can buffer against the harms of stigma and promote resiliency (Luoma et al., 2008; Somohano and Bowen, 2022; Wong et al., 2019). However, sparse research to date has elucidated the specific mechanisms through which MBIs address stigma and shame, nor has any research sought to identify for whom these mechanisms are most salient.

### Aims of the current paper

To this end, the current manuscript introduces the Mindful Resiliency in Recovery Model (MRRM), a novel framework that seeks to explain why MBIs may be particularly effective for marginalized individuals and those experiencing stigma, discrimination, and shame in the context of SUD. The MRRM builds upon current frameworks of stigma and recovery from SUD and extends these frameworks to individuals with stigmatized identities. It aims to describe a model that highlights the unique effectiveness of MBIs for stigmatized and marginalized individuals by fostering resilience to stigmatizing narratives. The MRRM represents a novel integration of existing theories, specifically tailored to address the unique challenges faced by marginalized and stigmatized individuals with SUD. Leveraging these established theories, the MRRM presents several significant advancements that surpass these individual theories when considered in isolation. As will be described in the following sections, the synthesis of these otherwise discrete theories guides the clinical application of MBIs for marginalized individuals while simultaneously identifying research opportunities that advance and clarify our understanding of the unique effectiveness of MBIs for marginalized and stigmatized groups.

It is important to note that terms like MBIs, third-wave treatments, and mindfulness and acceptance-based treatments are often conflated and, at times, differentiated from one another (Dimidjian et al., 2016; Hayes, 2004). For the purposes of the MRRM, the focus is not on the specific form of any given treatment manual, but rather on the functional processes which define it. In contrast to treatments like cognitive-behavioral therapy, the MRRM centers on treatments that emphasize changing one’s *relationship* to present moment experience, rather than changing the *content* of present moment experience. This distinction is best articulated by Crane et al. (2017) who highlight the essential components of MBIs in treating psychological disorders. As such, their definition of the core components comprising MBIs will be adopted here.

The following sections will explore the MRRM in detail, discussing its theoretical foundations, key premises, and implications for clinical practice and research. The MRRM aims to provide a comprehensive framework for understanding how MBIs can promote resiliency and recovery among individuals with SUD, especially those facing heightened stigma and marginalization. In doing so, it aims to contribute to the growing body of literature on MBIs for SUD to support the development of more effective, equitable, and contextually sensitive interventions for all communities.

## The mindful resiliency in recovery model (MRRM)

### Theoretical foundations

The MRRM integrates theories of mindfulness, stigma, and the role of contextual factors to explain how MBIs can support marginalized individuals in achieving recovery from SUD. As a novel integrated framework, it elucidates how mindfulness training can mitigate the adverse effects of stigma, enhance psychological resilience, and facilitate sustained recovery among those relegated to the margins of any given society or culture.

Two key theories of mindfulness, the Mindfulness-to-Meaning Theory (Garland et al., 2015) and the Monitor and Acceptance Theory (Lindsay and Creswell, 2017), provide insight into the core processes through which MBIs may promote recovery and resilience. The Mindfulness-to-Meaning Theory posits that mindfulness facilitates the generation of eudemonic meaning (i.e., meaning conducive to happiness/wellbeing), particularly in the face of adversity, through the mechanisms of decentering, reappraisal, and savoring (Garland et al., 2015). Decentering—akin to “self-as-context” in acceptance and commitment therapy (Hayes et al., 2013)—is the metacognitive state by which one disidentifies with sensory, cognitive, or emotional phenomena to achieve a reflexive distance in relation to internal experiences (Garland et al., 2015). In other words, it allows individuals to view thoughts as just thoughts, emotions just as emotions, and bodily sensations as bodily sensations without necessarily applying inherent meaning or veracity to them. Following decentering, the accompanying process of reappraisal allows for an adaptive reframing of one’s experience via an intentional shift in perspective (Garland et al., 2015). This reappraisal can further support a process of savoring whereby one’s ability to attend to and appreciate nourishing present-moment experiences is enhanced (Garland et al., 2015; Garland and Fredrickson, 2019).

**Figure 1:**
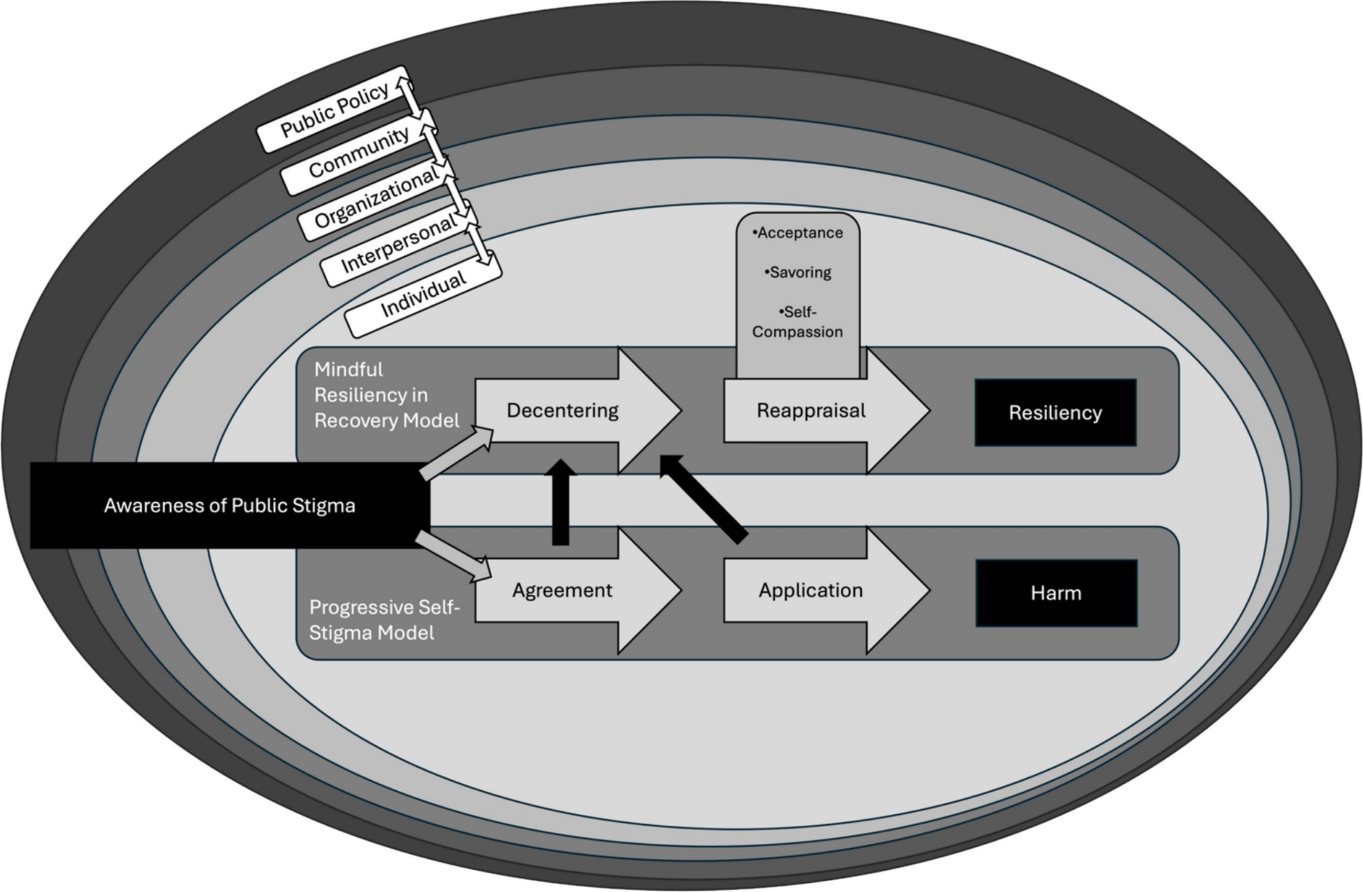
Visual representation of the mindful resiliency in recovery model (MRRM). At the center of the circle are two progressive paths: (1) the MRRM (path 1); (2) the Progressive Self-Stigma model (path 2). Each path represents the progression from awareness of public stigma to either (1) resiliency via the MRRM or (2) harm via the internalization of public stigma as described in the Progressive Self-Stigma model. Above the reappraisal process are additional mindfulness processes that support resiliency following decentering from stigmatizing public narratives. Note that the opportunity to decenter from the internalization of self-stigma is possible at any stage of the progressive self-stigma model as represented by the black arrows leading from path 1 to path 2. Further note how path paths 1 and 2 occur at the level of the individual, as represented by the inner-most oval (“Individual”). This inner-most oval is further surrounded by concentric ovals representing additional interdependent levels of context.

Parallel to this, the Monitor and Acceptance Theory highlights the core processes of attention monitoring and acceptance as central to the efficacy of MBIs generally (Lindsay and Creswell, 2019; Lindsay and Creswell, 2017). The Monitor and Acceptance Theory proposes that attention monitoring heightens awareness of present-moment experiences whereas acceptance supports an adaptive modification to one’s relationship to the present moment (Lindsay and Creswell, 2017). While studies seeking to deliberately test both the Monitor and Acceptance Theory and Mindfulness-to-Meaning Theory are ongoing, presently, there exists a promising amount of literature supporting their utility in explaining the essential processes underlying MBIs (Sgherza et al., 2022; Simione and Saldarini, 2023; Wang et al., 2023). Yet, both theories tend to be examined in isolation from one another, without attempts to integrate the two explicitly. The MRRM is novel in that it proposes a framework by which both the Mindfulness-to-Meaning Theory and Monitor and Acceptance Theory are indeed synthesized to further explain the processes underlying the efficacy of MBIs. Specifically, the MRRM highlights how each set of processes—decentering, reappraisal, and savoring from the Mindfulness-to-Meaning Theory, and attention monitoring and acceptance from the Monitor and Acceptance Theory—work in concert to support individuals in disengaging from negative self-referential thoughts, reinterpreting their experiences more adaptively, and cultivating a non-reactive, present-focused awareness that enhances their capacity to cope with, recover from, and thrive in the face of the stigmatizing narratives. An additional unique contribution of the MRRM lies in its contextualization of mindfulness processes within the lived experiences of marginalized individuals. It proposes that the salience and efficacy of specific mindfulness processes may vary based on individual and contextual factors (see Figure 1), a consideration not fully explored in existing theoretical models.

Alongside both the Monitor and Acceptance Theory and Mindfulness-to-Meaning Theory, the MRRM further integrates existing models of internalized stigma, namely the Modified Labeling Theory (Link et al., 1989) and Progressive Self-Stigma Model (Corrigan et al., 2011), to articulate the role of MBIs in fostering resilience in the face of stigmatizing social narratives. The Modified Labeling Theory proposes that individuals internalize societal beliefs (i.e., public stigma) of psychological disorders like SUD. It further articulates how the internalization of public stigma leads to detrimental self-preservation strategies like withdrawal, isolation, and self-denigration (Link et al., 1989). Extant research on the Modified Labeling Theory has demonstrated how public stigma can indeed lead to the internalization of stigma (Bos et al., 2013; Fox and Earnshaw, 2023; Glass et al., 2013). The Progressive Self-Stigma model builds upon this by delineating four distinct and progressive steps whereby public stigma results in individual harm via its internalization: (1) awareness of a stigmatizing narrative in the culture or society; (2) agreement with this narrative; (3) application of this narrative to oneself; (4) and harm as a product of applying the narrative to oneself (e.g., shame, self-contempt, isolation, etc.) (Corrigan et al., 2011). Leveraging these two frameworks, the MRRM highlights how MBIs punctuate and disrupt the progressive internalization of stigma via the mechanisms identified in the Mindfulness-to-Meaning and Monitor and Acceptance Theories. By fostering processes that enhance awareness without judgment and identification, and encourage adaptive reappraisal (e.g., self-compassion), MBIs are uniquely positioned to interrupt the internalization of public stigma. Further, even when stigma has been internalized, the MRRM highlights how these same processes found within MBIs can allow for the conscious abandonment of internalized stigma (i.e., decentering) in favor of more adaptive narratives (i.e., reappraisal; see Figure 1).

As a framework, the MRRM recognizes that the internalization of public stigma occurs within dynamic, multi-level contexts that influence recovery. Indeed, every human behavior is contextually bound, with contextual factors facilitating or hindering the reinforcement of behavior often outside the direct awareness of the individual (Burke et al., 2009; Bandura, 2006; Bouton, 2014; Witherington, 2017). Thus, when considering the core mechanisms underlying the efficacy of MBIs, and their effects on the progression and consequences of internalized stigma, it is essential to account for the ever-present nature of contextual factors that shape individual SUD recovery trajectories (Acuff et al., 2023; Tucker and Witkiewitz, 2021; Vuchinich et al., 2023). The MRRM draws upon the Dynamic Behavioral Ecological Model of Recovery (Witkiewitz and Tucker, 2024) and the Health Stigma and Discrimination Framework (Stangl et al., 2019) to articulate how MBIs support resiliency both at the individual level and at various levels of broader social-ecological contexts. Given the non-linear and dynamic nature of recovery and stigma internalization, as illuminated by the synthesis of these two frameworks, the MRRM seeks to clinically leverage and further compel research to study the flexible processes unique to MBIs that promote personalized adaptive responses to stigma-laden contexts (Moniz-Lewis et al., 2024). The MRRM further accounts for the interconnectedness between individual behavior and environmental factors (e.g., interpersonal, community, and policy factors) in the progression of internalized stigma. Including these contextual theoretical frameworks in the foundation of the MRRM alongside those previously discussed (i.e., theories of MBIs and stigma) enables the MRRM to remain theoretically coherent when accounting for the variability found across individuals and social-ecological contexts. Specifically, the MRRM accounts for this inter- and intra-contextual variability by postulating that the salience of MBI processes in promoting resilience to stigma can vary widely among individuals due to these contextual factors.

By synthesizing these theories, the MRRM offers a comprehensive framework for understanding how MBIs uniquely support individuals with SUD facing heightened stigma. Mindfulness processes like decentering, reappraisal, savoring, attention monitoring, and acceptance can buffer against the detrimental effects of self-stigma. While models like the Modified Labeling Theory focus primarily on individual level interventions, the inclusion of broader contextual frameworks within the MRRM allows for a nuanced understanding of the development of stigma-related harms for marginalized and stigmatized individuals with SUD. Thus, this framework better articulates the complex interplay between individual experiences and the social-ecological contexts shaping recovery (Witkiewitz et al., 2020).

The MRRM’s unique contribution lies in its contextualization of established mindfulness processes among marginalized individuals. It proposes that the salience and effectiveness of specific mindfulness processes may vary based on individual and contextual factors—a consideration not fully appreciated in existing models. Furthermore, the MRRM recognizes the fluidity of any individual’s social-ecological context and the dynamism inherent within it. As described in the core premises below, this model offers a theoretical expansion on existing mindfulness and stigma theories well-suited for future empirical investigation. Concurrently, it provides a practical framework for clinicians seeking to implement MBIs in a personalized and contextually sensitive manner that aligns with the complex realities of those we serve and would otherwise be less clear were these theories kept independent.

### Core premises of the MRRM

Building upon this theoretical foundation, the MRRM proposes three core premises that articulate how MBIs may promote resilience and recovery among marginalized and stigmatized individuals with SUD. These premises focus on the role of mindfulness training in reducing shame and enhancing resilience, the mediating effects of specific mindfulness processes on recovery, and the impact of mindfulness training on individuals’ relationships with their evolving social-ecological contexts. Each premise contains specific hypotheses that outline the expected relationships among these factors, the mechanisms therein, and their association with one’s social-ecological context. Through these premises, the MRRM holds the two-fold aim of both (a) providing a catalyst for future research that seeks to clarify and leverage the unique potential of MBIs to support the recovery among marginalized individuals with SUD who experience stigma, and (b) highlighting discrete mechanisms and treatment kernels to be targeted by clinicians utilizing MBIs among these populations.

Each premise, along with its accompanying hypotheses, is presented below. Importantly, while these premises seek to provide added theoretical guidance for MBI clinicians, the premises and hypotheses of the MRRM are preliminary and thus warrant further empirical investigation. Nevertheless, they are presented here to inform and inspire future research, thereby advancing our understanding of the potential of MBIs in supporting recovery among marginalized individuals with SUD.

#### Premise 1. Mindfulness training reduces the harmful psychological and emotional impacts of stigma on individual wellbeing by enhancing resilience and promoting recovery

Hypothesis 1a. Individuals who are marginalized and/or experience stigma will show greater improvements in wellbeing and recovery-related outcomes following mindfulness training, potentially exceeding those achieved with non-third wave treatments.

Hypothesis 1b. The unique benefit of MBIs among stigmatized individuals is in part due to the effects of mindfulness training on buffering against the harms of stigma, such that reductions in internalized stigma and shame will mediate the association between mindfulness training and improved recovery outcomes.

##### Evidence for premise 1

While preliminary, there is indeed evidence supporting Premise 1 of the MRRM. For example, a recent study by Joseph et al. (2023) conducted secondary analysis of a randomized controlled trial comparing mindfulness-based relapse prevention to standard cognitive-behavioral relapse prevention among legal-system-involved women with SUD. They found evidence for significant reductions in internalized shame (a construct closely related to internalized stigma) across treatment conditions, with those assigned to mindfulness-based relapse prevention reporting the greatest reductions. However, it is important to note that while these findings suggest potential advantages of MBIs, the differences were not statistically significant when compared to the non-third-wave comparison group. These results are mirrored by a more recent study that found support for a mindfulness-based psychoeducation program in reducing internalized stigma among adults with SUD (Mehel Tutuk and Budak, 2023). In this study, it was shown that mindfulness-based psychoeducation resulted in a significant reduction in internalized stigma and substance use proclivity among individuals with SUD. Specifically, the study found significant improvements in the subfacets of a measure of internalized stigma, including alienation, stereotype endorsement, discrimination experience, and social withdrawal (Mehel Tutuk and Budak, 2023). However, this study did not specific an active control group. Nonetheless, the results affirm that MBIs may reduce the psychological effects of stigma and improve the overall wellbeing of stigmatized individuals. This provides initial support for Hypotheses 1a and 1b and adds to the evidence showing the beneficial effects of mindfulness in aiding recovery from SUD by reducing stigma.

Additionally, research on MBIs with marginalized youth populations provides further support for Premise 1. Murray et al. (2018) conducted a literature review examining MBIs for youth involved in the criminal legal system—a highly marginalized and stigmatized group. The review synthesized findings from 10 studies and found that participation in MBIs was associated with significant reductions in perceived stress and improvements in emotional self-regulation and anger management. While these studies did not specifically measure internalized stigma or shame, the improvements in stress reduction and self-regulation suggest that mindfulness training reduces harmful psychological impacts of stigma and promotes wellbeing among marginalized youth, aligning with Hypotheses 1a and 1b.

Another study by Gul and Aqeel (2021) examined the efficacy of an acceptance and commitment therapy intervention targeting shame and stigma among individuals with SUD. They found that relative to those in a treatment as usual condition, those who received mindfulness training via acceptance and commitment therapy demonstrated significant decreases in stigma from pre- to post-course, as well as significant decreases in shame from pre- to post-course, and at follow-up. Further, those who received mindfulness training demonstrated improvements in general mental health, quality of life, psychological flexibility, and social support. Similarly, Ghaleh Emamghaisi and Atashpour (2020) examined the efficacy of acceptance and commitment therapy in reducing shame among males who use methamphetamine. Again, it was demonstrated that those who received mindfulness training via acceptance and commitment therapy experienced significant reductions in shame compared to a control condition.

These findings are further supported by Luoma et al. (2012) who found that those who received mindfulness training via an acceptance and commitment therapy-based intervention reported smaller immediate gains in shame initially before reporting larger reductions at follow-up. Those in the intervention group also reported fewer days of substance use and greater treatment attendance at follow-up with the effects of the intervention on treatment attendance being mediated by post-treatment levels of shame. These results highlight that shame may be an important mediator in the association of mindfulness-based treatment with SUD outcomes like treatment attendance. Further, it demonstrates that approaches targeting shame that leverage mindfulness processes appear to not only reduce levels of shame but further lead to improved treatment attendance and reduced substance use among those with SUD. These findings are further bolstered by research that has demonstrated an inverse correlation between levels of mindfulness and shame-oriented cognitions, both generally (Proeve et al., 2018; Woods and Proeve, 2014) and among individuals with SUD more specifically (Luoma et al., 2019; Brem et al., 2017). Taken together, this body of research demonstrates moderate preliminary support for Premise 1 of the MRRM, highlighting how MBIs have the potential to alleviate the internalization of stigma and shame that frequently hinder SUD treatment engagement.

#### Premise 2. Mechanisms specific to mindfulness training found within MBIs buffer against the harms of stigma and promote resiliency in recovery

Hypothesis 2a. Mechanisms of change identified in leading theories of MBIs—specifically decentering, reappraisal (e.g., self-compassion), savoring, present moment awareness, and acceptance will mediate the relationship between MBIs and reductions in internalized stigma and shame.

Hypothesis 2b. Contextual factors will moderate this mediation effect, such that these specific mechanisms will vary based on an individual’s social-ecological context. For example, the mediating effects of reappraisal may be more moderated by one’s current recovery capital.

##### Evidence for premise 2

While nascent literature has examined mechanisms leading to the efficacy of MBIs in targeting shame and self-stigma, there is nonetheless preliminary evidence supporting Premise 2 of the MRRM (Luoma et al., 2008; Stynes et al., 2022; Somohano and Bowen, 2022; Luoma et al., 2012; Harris, 2015; Masuda et al., 2009; Vowles et al., 2014). Indeed, the notion that specific mechanisms inherent to MBIs mediate the association between mindfulness training and reductions in internalized stigma and shame among individuals with SUD seems highly plausible based on this literature.

For example, a recent systematic review that explored the effectiveness of MBIs in addressing self-stigma and shame evidenced numerous potential mechanisms identified in this literature (Stynes et al., 2022). Notably, most of the studies in the review were not specific to SUD but nonetheless identified transdiagnostic processes theoretically shared across MBIs and psychological disorders with similar etiological mechanisms (e.g., eating disorders) (Dimidjian et al., 2016; Hayes, 2004; Kotov et al., 2021; Michelini et al., 2021). For example, enhancing psychological flexibility, emotion regulation, and acceptance were identified as salient mechanisms of change that mediate the association of treatment and reductions in self-stigma and shame (Stynes et al., 2022; Luoma et al., 2012; Lillis et al., 2009; Neacsiu et al., 2018). Further research evidences additional mechanisms such as increased awareness, decentering, and self-compassion as core mechanisms underlying the efficacy of MBIs in reducing shame and self-stigma (Somohano and Bowen, 2022; Harris, 2015; Ellerbroek et al., 2023; Heath et al., 2018; Moore et al., 2020; Price et al., 2020; Price and Smith-DiJulio, 2016). In a pilot study of a novel MBI for women with co-occurring SUD and post-traumatic stress disorder, mixed-methods analyses demonstrated support for numerous mechanisms that mitigate the harms of stigma (Somohano and Bowen, 2022). In this study, many women reported gains in present-moment awareness, non-judgmental acceptance, and self-compassion that facilitated an increased ability to adaptively respond to negative affective states like shame (Somohano and Bowen, 2022). Similarly, in another study examining mindfulness-based relapse prevention for SUD, qualitative analysis of participant experiences demonstrated that for many individuals bringing awareness and acceptance to negative affective states, such as shame resulting from stigma, was an essential process underlying its feasibility, acceptability, and efficacy (Harris, 2015). Further, Luoma et al. (2008) evidenced support for decreased experiential avoidance (i.e., acceptance) as a process underlying the efficacy of acceptance and commitment therapy for adults with SUD. Similar findings were demonstrated in a recent study of a mindfulness and self-compassion-based intervention for adults with opioid use disorder (Moore et al., 2022). The study found that participants who underwent the MBI program demonstrated significant improvements in self-compassion from baseline to 24 weeks. Qualitative interviews revealed that participants attributed these improvements to increased present-moment awareness, self-kindness, and a greater sense of common humanity (Moore et al., 2022). Moreover, the review by Murray et al. (2018) identified improved self-regulation, stress reduction, and anger management as key outcomes of MBIs for legal system-involved youth. These mechanisms align with Hypothesis 2a, suggesting that mindfulness processes such as emotion regulation and present-moment awareness mediate the relationship between mindfulness training and reductions in psychological distress among marginalized youth. While preliminary, these findings align with Hypothesis 2a as they illustrate how mechanisms of MBIs, like self-compassion, present-moment awareness, and acceptance underly its effect in reducing internalized stigma and shame, thereby promoting recovery and resilience in individuals with SUD.

While the available evidence lends preliminary support to Hypothesis 2a, to date there is limited direct evidence for Hypothesis 2b, which posits that contextual factors moderate these mechanisms. However, the lack of evidence is not due to a lack of findings on the moderation of contextual factors but rather a lack of investigation into and appreciation for context altogether (Moniz-Lewis et al., 2024). As discussed elsewhere, the field of substance use research has largely overlooked the role of contextual factors in the recovery process, with few existing studies examining how factors such as socioeconomic status, cultural background, recovery capital, and social support might influence the effectiveness of MBIs through these mechanisms (Witkiewitz and Tucker, 2024; Tucker and Witkiewitz, 2021; Moniz-Lewis et al., 2024; Moniz-Lewis, 2024). For example, in a recent systematic review on mechanisms of change in MBIs for SUD, only 3 out of 22 studies that met the criteria for the review attended to contextual factors in any way, with no studies looking at the moderation effects of contextual factors impacting these mechanisms (Moniz-Lewis, 2024). Arguably, this lack of investigation is not merely an oversight but rather a side-effect of a predominant worldview and philosophy of science that discounts the complexities, contexts, and lived experiences of individuals from marginalized backgrounds (Burlew et al., 2021; Buchanan et al., 2021).

While there is a dire need to both investigate and appreciate the role of context in MBI research more broadly, there is nonetheless strong evidence to suggest the potential moderating roles of contextual factors in SUD recovery (Spears, 2019; Moniz-Lewis et al., 2024). For example, it has been shown that proximity to substance use outlets, financial stability, access to recovery capital (e.g., supportive social networks), and regional income equality are predictive of maintenance of recovery over time (Swan et al., 2021; Joshi et al., 2022; Pouille et al., 2021; Slutske et al., 2019). Given these findings, it is reasonable to hypothesize that contextual factors may moderate not only access to MBIs but also the efficacy of specific mindfulness mechanisms in promoting recovery from SUD—hence a moderated mediation model of contextual factors influencing mindfulness processes.

The MRRM hypothesizes that the effectiveness of mindfulness processes (e.g., reappraisal, decentering, etc.) in reducing internalized stigma and shame may vary depending on an individual’s social-ecological context. For instance, individuals in environments characterized by less stigma and greater support for recovery (e.g., non-discriminatory drug policies, access to substance-free activities, and high recovery capital) may experience enhanced benefits from mindfulness processes like reappraisal as adaptive reappraisal is reinforced by external supports. Conversely, in highly stigmatizing environments (e.g., discriminatory drug policies, legal-system involvement, incarceration, etc.), mechanisms like self-compassion may prove more crucial as individuals must rely more on internal resources to find resiliency through adversity.

This proposition aligns with ecological models of health behavior which recognize the dynamic interplay between individual health behaviors and environmental contingencies (Stangl et al., 2019). However, it is important to note that these propositions remain speculative and require empirical further investigation. Hypothesis 2b thus emphasizes the need to move beyond a context-insensitive approach to understanding mindfulness processes in SUD recovery by calling for research that explicitly examines how varied social-ecological factors may moderate the mediating effects of specific mindfulness mechanisms. Such work is critical to validate, refute, or refine Hypothesis 2b and to identify for whom and in what context a given mechanism is effective in buffering against stigma.

#### Premise 3. Mindfulness training bolsters individuals’ engagement with their evolving social-ecological environment, contributing to sustained improvements in recovery over time

Hypothesis 3. Those who receive mindfulness training will be more likely to experience improvements in their social-ecological contexts (e.g., social support, recovery capital, interpersonal relationships, etc.) which in turn will predict improved long-term recovery outcomes.

##### Evidence for premise 3

The importance of social factors in recovery is well documented through recent research on social recovery capital, defined as the extent to which an individual’s social networks support SUD recovery (Gu et al., 2023; Cano et al., 2017; Cloud and Granfield, 2008). A growing body of research supports the notion that mindfulness training can positively impact individuals’ social-ecological contexts, including social recovery capital, which in turn contributes to improved recovery outcomes for those with SUD (Gu et al., 2023; Gannon et al., 2022; LaBelle et al., 2023; Skoranski et al., 2019; Smith et al., 2020; Thiermann and Sheate, 2022). This premise is grounded in the understanding that recovery occurs within a dynamic social-ecological context, where various factors at different levels of context (e.g., individual, interpersonal, community, and public policy) interact to influence recovery processes (Stangl et al., 2019; Witkiewitz and Tucker, 2024). Indeed, research demonstrates that the benefits of mindfulness training extend beyond the individual to encompass broader contextual factors (LaBelle et al., 2023; Adair et al., 2018; Lindsay et al., 2019). Further, research on discriminatory contexts consistently demonstrates how experiences like racism, stigmatization, and discrimination can lead to isolation, withdrawal, and decreased wellbeing generally (Amaro et al., 2021; Brondolo et al., 2012). As such, an important factor explaining the efficacy of MBIs in buffering against stigma may be found in how mindfulness training changes one’s relationship with their social-ecological context.

Preliminary research suggests that social support and interpersonal functioning are key factors influenced by mindfulness training and impact one’s broader SUD recovery context. Birtel et al. (2017) found that perceived social support improved mental health outcomes among individuals with SUD and was mediated by reductions in internalized stigma and shame. Though not directly examining an MBI, when discussing their findings, the authors note that “targeting the stigma of substance [use] may not only reduce the negative psychological impact of stigma on people’s appraisals of their emotions, cognitions, and behaviors, but may also help people to utilize their social support in order to enhance their coping strategies to deal with both their primary illness as well as the stigma attached to it” (Birtel et al., 2017). Qualitative evidence from a previously discussed study (Moore et al., 2022) further supports this proposition for MBIs specifically. As mentioned, participants with opioid use disorder who engaged in mindfulness and self-compassion training reported enhanced awareness and prosocial behavior in their daily lives as a product of the training. Importantly, participants reported experiencing enhanced positive social support and engagement in their relationships following the training, which bolstered their recovery. Participants reported on the processes of enhanced mindfulness and self-compassion as being core to this shift in their relationship with their social-ecological context. A more recent study by LaBelle et al. (2023) provides additional support for the uptake of mindfulness skills leading to enhanced recovery capital. Specifically, among members of a Buddhist-based peer support program for addiction recovery, the study found that levels of mindfulness, frequency of mindfulness practice, and perceived support from peers significantly predicted recovery capital. Notably, the frequency of meditation practice was a better predictor of recovery capital than the duration of meditation practice (LaBelle et al., 2023). This suggests that consistent, regular engagement with mindfulness practices may be particularly beneficial for building and maintaining the underlying processes (e.g., acceptance, self-compassion, etc.) that lead to improved prosocial engagement with one’s recovery context. Findings such as these underscore the potential of mindfulness training to not only improve individual-level factors but also to subsequently enhance the quality of interpersonal relationships and social support networks as a result of the enhancement in these individual-level factors.

While these studies lend preliminary support for the role of mindfulness processes in enhancing social support and building recovery capital, it is important to note that research directly examining the impact of mindfulness training on broader social-ecological contexts in SUD recovery remains nascent. Therefore, there is a need for research that more explicitly tests Premise 3 to better glean how MBIs influence individuals’ relationships with their recovery context despite stigmatizing narratives.

### Implications for stigmatized, marginalized, and underserved populations

Marginalized and underserved communities, who often face multiple, intersecting forms of stigma and discrimination, experience disproportionate harms from SUD relative to more dominant groups (Alegria et al., 2011; Greene, 2023; Room, 2005; Birtel et al., 2017). The compounded effects of societal marginalization and SUD-related stigma can exacerbate health disparities, impede access to quality care, and undermine recovery efforts (Lê Cook and Alegría, 2011; Van Boekel et al., 2013; Douglass et al., 2023; Morris and Schomerus, 2023). Addressing these disparities requires a multi-level approach beyond just individual-level efforts (Stangl et al., 2019; Gronholm et al., 2017). There is a dire need to change the political and social policies, practices, and narratives that perpetuate stigma and health disparities (Grisamore and DeMatteo, 2024; Koehm et al., 2024; Rehman et al., 2024). Alongside these concerted efforts to bring about greater social justice and compassion, there is a need to develop and implement contextually responsive, affirming, and efficacious interventions that attend to the unique needs and experiences of the individuals experiencing stigmatization and marginalization (Stangl et al., 2019; Mincin, 2018; Sibley et al., 2024). In other words, while social efforts are needed to address the root causes of stigma and marginalization at a social and political level, there is nonetheless a necessity to acknowledge and uplift the innate capacities of marginalized individuals and communities to remain resilient in the face of these social injustices. To that end, the MRRM offers a promising framework for identifying how MBIs may be particularly well-suited to this task by targeting key mechanisms of change that can help buffer against the harms of stigma and promote resilient recovery among marginalized individuals with SUDs.

Figure 1 provides a visual representation of the MRRM, illustrating how mindfulness processes promote resilience in the face of stigma. The diagram demonstrates the model’s unique integration of individual-level mindfulness processes within broader social-ecological contexts. At its center, two pathways diverge from the awareness of public stigma: one leading to resiliency through mindfulness processes, and the other to harm via internalized stigma. The concentric circles surrounding these two pathways represent the multiple interrelated levels of context - from individual to public policy—that remain largely beyond the scope of any given MBI yet nonetheless influence one’s recovery process. The figure further illustrates the possibility of decentering from stigmatizing narratives at any point in the processes of internalizing public stigma (i.e., the progressive-self stigma model). Further, as individuals engaged in the reappraisal process as outlined by the MRRM, additional complimentary mindfulness processes, namely acceptance, savoring, and self-compassion, can support the cultivation of innate resilience via reappraisal. This visual framework not only synthesizes the theoretical foundations of the MRRM but outlines a testable model seeking to explain how specific mindfulness processes may buffer against stigma when accounting for contextual factors. For instance, it suggests the reappraisal processes may contain differential effects depending on an individual’s position within these broader contexts, a proposition outlined by Hypothesis 2b which warrants further empirical investigation.

At the core of the MRRM lies the foundational assumption that all individuals, regardless of their race, gender, socioeconomic status, lived experiences, etc. have the innate capacity to define, discover, and lead lives of greater purpose, meaning, and wellbeing. Rooted in this assumption, the MRRM highlights key mechanisms that can be leveraged in the clinical practice of MBIs to support marginalized individuals in decentering from stigmatizing narratives and developing adaptive reappraisals of their life circumstances. Drawing upon the Mindfulness-to-Meaning Theory (Garland et al., 2015) and the Monitor and Acceptance Theory (Lindsay and Creswell, 2017), the MRRM posits that mindfulness enables individuals to become aware of and step back from the maladaptive appraisals by which public stigma becomes internalized. By instead entering a state of metacognitive awareness, the process of decentering allows for a broadening of attention and the adaptive reappraisal of one’s present moment experience—one that more accurately represents the innate capacities for wellbeing existent in all individuals.

For individuals with SUDs who come from diverse groups, these decentering and reappraisal processes could entail the acknowledgment of the innate strengths within oneself, one’s community, or one’s cultural practices, such that one’s potential for recovery is not defined by societal prejudices or negative stereotypes about addiction. Instead, one can begin to see oneself as a whole person with inherent dignity, capable of change, and connected to something greater than a limited, socially defined sense of self. This shift in perspective can be particularly empowering for marginalized individuals who may have internalized stigmatizing messages about their substance use and its implications for their identity, worth, and future opportunities. To maximize these benefits, MBIs can be adapted to explicitly integrate core mindfulness principles relevant to marginalized populations with contextual supports (Moniz-Lewis et al., 2024). For example, integrating cultural traditions, community values, and collective healing practices into mindfulness exercises can help individuals connect more deeply with their sources of innate resilience. Such adaptations not only provide a clear mediating link to specific populations but also align with the MRRM’s emphasis on contextualizing mindfulness processes within the lived experiences of marginalized individuals.

Again leveraging the Mindfulness-to-Meaning Theory, the MRRM emphasizes the role of savoring in enhancing the benefits of adaptive reappraisals (Garland et al., 2015). By mindfully attending to the positive aspects of their lives and communities, marginalized individuals in SUD recovery can tap into sources of meaning, connection, and strength that transcend their stigmatized status (Garland and Fredrickson, 2019; Garland, 2021). For example, one may savor moments of genuine connection with supportive others, experiences of personal accomplishment in their recovery journey, or the beauty and resilience they witness in their communities and culture. This savoring process can motivate values-driven behavior and the pursuit of a purposeful life, even in the face of adversity (Garland, 2021). It can help individuals build an adaptive recovery identity and cultivate hope for the future, countering the socially perpetuated stigma and shame associated with SUD (Luoma et al., 2019; Moore et al., 2020). MBIs may further support stigmatized individuals in extending kindness and understanding toward themselves, both decreasing the likelihood that public stigma becomes internalized or countering the harms of stigma if already internalized (Wong et al., 2019). By learning how to relate to oneself with compassion, even in the face of SUD-related challenges and setbacks, individuals can develop a more stable and affirming sense of self, reducing the likelihood of a return to undesired substance use in response to stigma-induced distress (Somohano and Bowen, 2022; Witkiewitz et al., 2020).

The MRRM also recognizes the importance of contextual factors, such as social support, interpersonal relationships, and access to recovery-supportive environments in buffering the impact of SUD-related stigma for marginalized individuals. No group or community is homogeneous, and as such, it is essential to acknowledge not only the moderating effects of context on individual behavior, but also the heterogeneous and varied pathways to recovery that exist for individuals from marginalized communities (Witkiewitz et al., 2020). To this end, the MRRM seeks to embrace contextual sensitivity to account for how contextual factors may impact the processes underlying the efficacy of MBIs for SUD. For example, mindfulness training, especially when conducted in group settings, may enhance marginalized individuals’ sense of belonging and social support (Greenfield et al., 2018; Spears, 2019; Witkiewitz et al., 2013; Foale et al., 2024). Participating in an MBI group with others who share similar experiences can help normalize individual struggles and reduce isolation (LaBelle et al., 2023; Russo, 2019). Furthermore, by fostering reappraisal processes such as self-compassion and forgiveness, MBIs may facilitate more adaptive responses to stigma-related interpersonal transgressions (Wong et al., 2019; Luberto et al., 2018; Miyagawa and Taniguchi, 2022; Neff, 2023). For instance, individuals may learn to accept the fears and misconceptions that underlie others’ stigmatizing beliefs, allowing for greater empathy and less personalization of negative interactions.

The premises offered by the MRRM have additional implications for clinical practice with stigmatized and marginalized populations seeking SUD treatment beyond those mentioned above. When adapting MBIs for these groups, it may be particularly beneficial to emphasize practices that cultivate self-compassion, reappraisal skills, and savoring (Moniz-Lewis et al., 2024). For example, loving-kindness meditations and self-compassion exercises can be incorporated to help individuals develop a more caring and forgiving stance toward themselves and build greater self-efficacy in being able to achieve self-defined recovery (Witkiewitz and Tucker, 2024; Chen, 2021; Hofmann et al., 2011; Moniz-Lewis et al., 2022). Cognitive reappraisal strategies can be explicitly taught and practiced, with a focus on identifying and challenging SUD-related stigmatizing beliefs (Luoma et al., 2008; Mehel Tutuk and Budak, 2023). Savoring practices found in MBIs like mindfulness-oriented recovery enhancement can be leveraged to support marginalized individuals in attending to the nourishing aspects of their lives and counteract the predominance of stigma-related self-perceptions (Garland, 2013; Bryan et al., 2022). Fostering greater awareness of stigma and its impact on SUD recovery can support increased self-acceptance and foster a sense of common humanity (Garland and Fredrickson, 2019; Chen, 2021; Shreffler et al., 2022). Further, to enhance the effectiveness of MBIs for marginalized communities, it may be beneficial to adapt these interventions by explicitly linking core mindfulness mechanisms and concepts to contextually relevant experiences. For example, this could involve tailoring reappraisal practices directly to culturally specific strengths and including collaborative discussions of how mindfulness practices can be used when experiencing discrimination or stigma generally (Moniz-Lewis et al., 2024). By doing so, the MRRM outlines direct avenues by which MBIs can be tailored to strengthen the underlying mechanisms that allow marginalized individuals to benefit from their innate resiliency when experiencing stigma. Indeed, cultural adaptions of existing MBI programs exist (Castellanos et al., 2020), and while arguably not widely yet implemented these programs offer clear examples of how MBIs can be improved to better serve those from marginalized groups.

Ultimately, the MRRM postulates that MBIs have the potential to empower marginalized and stigmatized individuals with SUDs to transcend limiting narratives and actualize their innate capacity for wellbeing. By developing decentered present-moment awareness, adaptive reappraisal (e.g., self-compassion, savoring, etc.), and a non-reactive values-driven behavior, individuals can prevent or overcome the internalization of stigma. The MRRM provides a framework for understanding and optimizing the mechanisms through which MBIs confer these benefits. Further, it offers guidance on the application of more efficacious interventions to be implemented alongside efforts to uproot systematic causes perpetuating the stigmatization of individuals with SUD.

## Discussion

The current manuscript introduces the MRRM, a novel framework that seeks to explain why MBIs may be particularly effective for marginalized individuals with SUD who experience stigma and discrimination. The MRRM draws upon existing theories of mindfulness, stigma, and contextual factors in recovery to propose an integrated theoretical framework elucidating how mindfulness training can mitigate the adverse effects of stigma, enhance psychological resilience, and facilitate sustained recovery among those unjustly relegated to the margins of society.

At its foundation the MRRM synthesizes core theoretical models: the Mindfulness-to-Meaning Theory (Garland et al., 2015), the Monitor and Acceptance Theory (Lindsay and Creswell, 2017), the Modified Labeling Theory (Link et al., 1989), the Progressive Self-Stigma Model (Corrigan et al., 2011), the Dynamic Behavioral Ecological Model of Recovery (Witkiewitz and Tucker, 2024), and the Health Stigma and Discrimination Framework (Stangl et al., 2019). Through integrating these theories and frameworks, the MRRM articulates how core mindfulness processes found within MBIs buffer against the detrimental effects of self-stigma and promote resilience in the face of stigmatizing narratives. Further, it seeks to extend current work on the processes underlying the efficacy of MBIs to account for *whom and in what contexts* these processes are salient. In doing so, it offers a theoretical model that accounts for recent evidence suggesting a unique efficacy of third-wave treatments for individuals from marginalized groups (Dela Cruz et al., 2022; Greenfield et al., 2018; Spears, 2019; Sun et al., 2022; Witkiewitz et al., 2013), while further accounting for the role of contextual factors in moderating recovery outcomes (Witkiewitz and Tucker, 2024; Tucker and Witkiewitz, 2021).

The MRRM has significant clinical and research implications for those serving stigmatized, marginalized, and underserved populations with SUD. Clinically, the MRRM highlights the opportunity for MBIs to support marginalized individuals in decentering from stigmatizing narratives and developing adaptive reappraisals of their life circumstances, tapping into innate resources and strengths, both within the individual and their surrounding context, that transcend stigmatizing social narratives. The MRRM offers guidance when adapting MBIs for these populations, highlighting practices that target core change processes which may be especially salient in the recovery process (Moniz-Lewis et al., 2024; Venner et al., 2008).

From a research perspective, the MRRM offers a testable framework for investigating the mechanisms and contextual factors that contribute to the efficacy of MBIs among marginalized populations with SUDs. By proposing specific hypotheses related to the role of mindfulness processes in reducing internalized stigma, promoting resilience, and enhancing engagement with supportive social-ecological contexts, the MRRM provides a roadmap for future empirical work. Furthermore, the MRRM highlights the need for research on MBI adaptations tailored to the unique needs and experiences of marginalized communities, ensuring that these interventions are not only effective but also contextually sensitive. By guiding research efforts toward a more nuanced understanding of how MBIs work for whom and under what conditions, the MRRM can inform the development of more targeted and impactful interventions for marginalized individuals with SUDs.

## Limitations and future research

The MRRM is not without limitations that dually serve as opportunities for future research. Importantly, the MRRM proposes three core premises that outline how MBIs may uniquely support marginalized individuals with SUDs. Alongside these premises, the MRRM presents testable hypotheses that will advance our understanding of the efficacy of MBIs for marginalized communities, the role of MBIs in mitigating the harms of stigma and promoting resiliency, and the mechanisms therein. An important current limitation of this model is that the evidence supporting each premise and its accompanying hypotheses is preliminary. As such, the MRRM is presented as a theoretical model to be updated, refined, or refuted altogether as research on the efficacy of MBIs for marginalized populations advances. Thus, future research that tests each premise of the MRRM, and its hypotheses, is essential to advancing our understanding of how MBIs can support recovery among marginalized populations who experience stigma.

An important limitation of the MRRM is the lack of clear empirical evidence directly supporting some of its sub-hypotheses, particularly those related to the moderating effects of contextual factors on mindfulness mechanisms (e.g., Hypotheses 2b and 3). While the theoretical rationale is strong and preliminary studies offer preliminary support, the absence of substantial empirical studies testing these specific hypotheses limits the current applicability of the model. This lack of direct evidence is noteworthy and highlights the need for future research to empirically validate these propositions. Without such evidence, the model remains speculative in these areas, and its practical implications should be interpreted with caution.

It is crucial to note that the vast majority of research informing the MRRM was conducted in Western cultures, primarily in a United States American context. As such, the extent to which this model accounts for the potential efficacy of MBIs in non-Western contexts is unclear. Thus, there is an additional need for future research that examines the applicability and explanatory power of the MRRM in non-Western contexts. Consequently, there is a dire need for future research on MBIs that prioritizes the inclusion of participants from various cultural, ethnic, and socioeconomic backgrounds to better understand the efficacy and processes within MBIs for these groups (Spears, 2019; Wilson et al., 2017).

Future research should further prioritize the empirical validation of the MRRM in diverse samples and settings via methods rooted in a plurality of world views (Hayes et al., 1988). Thus, methods that leverage both qualitative and quantitative investigations of the hypothesized mechanisms of MBIs’ effects on stigma-related outcomes, as well as methods that allow for the merging of nomothetic and idiographic findings are essential next steps in this literature as a whole (Hayes et al., 2024; Molenaar, 2004; Piccirillo and Rodebaugh, 2019). Research on the efficacy and acceptability of MBI adaptations tailored to specific underserved communities will be essential in translating empirical findings to direct clinical practice such that it results in a tangible positive impact on the communities these treatments seek to serve (Spears, 2019; Moniz-Lewis et al., 2024; Wilson et al., 2017). Further, given the scarcity of studies that directly examine the efficacy of MBIs in targeting stigma when compared to non-third-wave treatments (e.g., cognitive-behavioral therapy), it will be important to examine the efficacy of MBIs compared to active control groups.

## Conclusion

In conclusion, the MRRM provides a novel integrated framework for better understanding and optimizing the mechanisms through which MBIs confer benefits to marginalized individuals with SUDs, specifically seeking to explain the role of stigma in the recovery processes. While the literature supporting the MRRM is preliminary, it nonetheless offers promise in empowering marginalized individuals to transcend limiting and stigmatizing social narratives and to instead actualize their innate resiliency and capacity for wellbeing. To this end, MBIs can play a vital role in uplifting marginalized individuals and mitigating the harms resulting from the disparities and social injustices that perpetuate SUD-related stigma. Through this work, we can advance the promise of MBIs as a means of promoting individual and collective wellbeing in the face of the pervasive and pernicious stigma surrounding SUDs. By empowering marginalized individuals to reclaim their narratives, actualize their innate resilience, and pursue lives of greater meaning, purpose, and wellbeing, MBIs can play a vital role in ameliorating the suffering resulting from stigma. Ultimately, this work is not only about alleviating individual suffering, but empowering all individuals, especially those that have been historically marginalized, stigmatized, and underappreciated, such that we all can collectively create a more compassionate and equitable world for all.

## Data Availability

The original contributions presented in the study are included in the article further inquiries can be directed to the corresponding author.
